# 5-mehtyltetrahydrofolate rescues alcohol-induced neural crest cell migration abnormalities

**DOI:** 10.1186/s13041-014-0067-9

**Published:** 2014-09-16

**Authors:** Yu Shi, Jiejing Li, Chunjiang Chen, Manzi Gong, Yuan Chen, Youxue Liu, Jie Chen, Tingyu Li, Weihong Song

**Affiliations:** Department of Clinical laboratory, Chongqing, China; Chongqing City Key Lab of Translational Medical Research in Cognitive Development and Learning and Memory Disorders, and Ministry of Education Key Lab of Child Development and Disorders, Children’s Hospital of Chongqing Medical University, Chongqing, 400014 China; Department of Biology, West Virginia University, Morgantown, WV USA; Townsend Family Laboratories, Department of Psychiatry, Brain Research Center, The University of British Columbia, 2255 Wesbrook Mall, Vancouver, BC V6T 1Z3 Canada

**Keywords:** Alcohol, 5-mehtyltetrahydrofolate, Neural crest, FASD, Xenopus

## Abstract

**Background:**

Alcohol is detrimental to early development. Fetal alcohol spectrum disorders (FASD) due to maternal alcohol abuse results in a series of developmental abnormalities including cranial facial dysmorphology, ocular anomalies, congenital heart defects, microcephaly and intellectual disabilities. Previous studies have been shown that ethanol exposure causes neural crest (NC) apoptosis and perturbation of neural crest migration. However, the underlying mechanism remains elusive. In this report we investigated the fetal effect of alcohol on the process of neural crest development in the Xenopus leavis.

**Results:**

Pre-gastrulation exposure of 2-4% alcohol induces apoptosis in Xenopus embryo whereas 1% alcohol specifically impairs neural crest migration without observing discernible apoptosis. Additionally, 1% alcohol treatment considerably increased the phenotype of small head (43.4% ± 4.4%, total embryo n = 234), and 1.5% and 2.0% dramatically augment the deformation to 81.2% ± 6.5% (n = 205) and 91.6% ± 3.0% (n = 235), respectively (P < 0.05). Significant accumulation of Homocysteine was caused by alcohol treatment in embryos and 5-mehtyltetrahydrofolate restores neural crest migration and alleviates homocysteine accumulation, resulting in inhibition of the alcohol-induced neurocristopathies.

**Conclusions:**

Our study demonstrates that prenatal alcohol exposure causes neural crest cell migration abnormality and 5-mehtyltetrahydrofolate could be beneficial for treating FASD.

## Background

Fetal alcohol spectrum disorders (FASD) results from maternal alcohol abuse and is featured by abnormal facial morphology, ocular anomalies, congenital heart defects, microcephaly and intellectual disability [1]. Dysregulation of neural crest (NC) development has been contributing to the majority of malformations. Studies using numerous animal models reveal that alcohol can initiate neural crest cell apoptosis, and eventually develops FASD [2-4].

NC cells are multipotent migratory cells originating at the ectoderm. After induction and specification, NC cells form many cell types ranging from the cranial bone, smooth muscle to peripheral and enteric neurons and glia [5-7]. NC development is tightly regulated by finely tuned gene regulatory network (GRN) and orchestrated hierarchically. NC induction is initialized at beginning of neurulation. Morphorgens such as Wnt, fibroblast growth factor (FGF), retinoic acid (RA) and bone morphogenetic protein (BMP) secreted from the paraxial mesoderm and epidermis regulates the expression of a group of transcription factors (*Pax3, Zic1, Msx1*) whereby defines the boarder of neural crest. Subsequently, these transcription factors stimulate the expression of the NC specification genes including *FoxD3*, *Slug* and *Twist* to determine the cell fate of NC [8,9]. Once NC induction is accomplished NC cells undergo the epithelial-mesenchymal transition (EMT) and eventually migrate along predetermined trajectories.

Given NC induction and migration occurs sequentially [10,11], induction associated neurocristopathies can be identified to reflect migration abnormality. Alcohol appears to be able to affect NC development via either interfering NC cell migration or prompting cell apoptosis [12] that is indistinguishable from induction defects. Folate deficiency has long been known to be involved in NC defects. Abnormal folic acid level leads to dysfunction of NC migration [11,13]. The disturbance of folic acid metabolism causes craniofacial malformation [14,15] and heart defects [16]. Furthermore, studies showed that supplementation of folic acid successfully modulates alcohol-induced gene expression profiles and alleviates the developmental defects [17,18]. However, the exact mechanism underlying on what specific developmental phase folic acid affects remains elusive.

In this study using Xenopus model system we investigated whether alcohol directly affected migration. We found that pre-gastrulation exposure of 2-4% alcohol induces developmentally non-specific apoptosis, whereas 1% alcohol specifically impairs neural crest migration without significant apoptosis, which consequently lead to neurocristopathies-like abnormalities. Furthermore, we showed that homocysteine, a molecular reported to affect neural crest migration [19], was elevated after alcohol exposure and 5-mehtyltetrahydrofolate (5MTHF) ameliorated endogenous homocysteine levels induced by alcohol exposure and dramatically rescued the impaired neural crest migration.

## Results and discussion

### Alcohol induces neurocristopathies in Xenopus *leavis*

**Figure 1:**
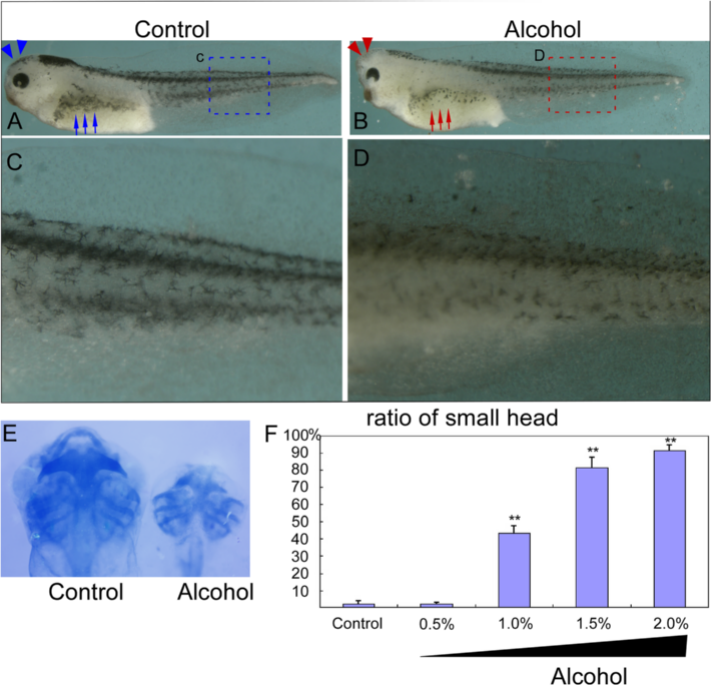
**Alcohol exposure impaired pigment and cranial cartilage formation. (A,C)** Control embryos show normal amount and pattern of pigment cells. **(B,D)** Alcohol treated embryos develop less and punctate pigment cells. **(E)** Alcohol treated embryos at stage 45 exhibited truncated cranial cartilage. Compared to control embryos, both Meckel’s and palatoquadrate were absent from treated embryos and the ceratohyal cartilages were markedly smaller. **(F)** The alcohol exposure deteriorates cartilage formation. Ratio of small to total heads exhibit no significant difference between control (2.3% ± 1.5%, n = 190) and 0.5% alcohol condition (2.3% ± 0.7%, n = 214). Additionally, 1% alcohol treatment considerably increased the phenotype of small head (43.4% ± 4.4%, total embryo n = 234), and 1.5% and 2.0% dramatically augment the deformation to 81.2% ± 6.5% (n = 205) and 91.6% ± 3.0% (n = 235) respectively (P < 0.01). At each concentration, the experiments were repeated 3 times, and n represents the total embryos of triplicate assay. The values represent means ± SEM. *p* < 0.01, by student T test.

To examine FASD in *Xenopus*, embryos are treated with 0-2% alcohol starting from pre-gastrulation (stage 9) and washed at late neural plate stage (stage 16) and then cultured in modified Barth solution (MBS). Since cartilage architecture and pigment cell patterning are the most prominent developmental structure of NC derivatives, we examined malformations of cartilage and pigment cell development at stage 45. Alcohol treatment resulted in deformations of the pigment (Figure 1B; red arrow and Figure 1D) and the head structure (Figure 1B; red arrow head) compared to control group (Figure 1A; blue arrow and Figure 1C). Alcohol exposure also led to aberrant cranial cartilage formation with a shrunk dysplasic size and the small head (Figure 1E). In terms of small head phenotype, there is no significant different between 0.5% alcohol (2.3% ± 0.7%, n = 214) and control group (2.3% ± 1.5%, n = 190) (P > 0.05). However, 1% alcohol significantly increased the phenotype of small head (43.4% ± 4.4%, n = 234), and 1.5% and 2.0% dramatically augment the deformation to 81.2% ± 6.5% (n = 205) and 91.6% ± 3.0% (n = 235) respectively (P < 0.01) (Figure 1F). The results indicate that alcohol destructs neural crest normal development in a dosage dependent manner in Xenopus model system.

### Alcohol causes neither neural crest induction nor cell apoptosis at neural plate stage

**Figure 2:**
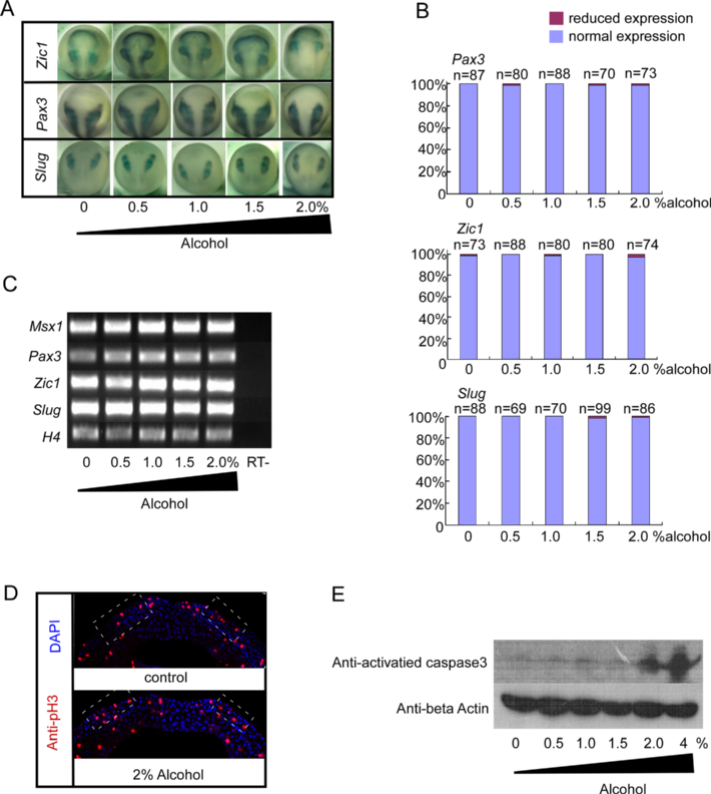
**Exposure to lower concentration of alcohol had no significant toxicity to neural crest development.** We harvested the embryos at stage 16 for Whole-mount In Situ Hybridization (WISH). **(A)** Both boarder determinator gene *Pax3*, *Zic1* and neural crest specification marker gene *slug* are kept intact. **(B)** Quantification of the effect of alcohol exposure on the expression of neural crest markers. **(C)** RT-PCR assays display expression of Msx1, Pax3, Zic1 and Slug are identical according to divergent alcohol treatment. **(D,E)** Alcohol less than 2% did not trigger abnormality of cell proliferation (labeled with pH3). Neural crest cell apoptosis was induced by 2% alcohol treatment as shown by activated caspase3. (normalized with beta actin).

The development of NC has two functionally connected but developmentally separated phases that involve induction and migration. FASD could be the consequence of dysregulating of either induction or migration. Previous studies mainly focused on characterizing its impact on NC migration. Here we investigated how alcohol specifically affected NC induction. Xenopus embryos were incubated with 0.5% to 2% alcohol at the onset of gastrulation (stage 9) and collected at neural stages (stage 16). An *in situ* hybridization was performed using the border markers *Pax3* and *Zic1* as well as NC marker *slug*. The expression patterns of all three genes were not affected during the differentiation of the pre-migratory neural crest (Figure 2A and B). RT-PCR assays were performed to further confirm the results. Consistent with the *in situ* hybridization data, the expression levels of the border genes *Msx1*, *Pax3* and *Zic1* and neural crest marker gene *Slug* showed similar intensity to those in control embryos (Figure 2C). These results suggest that alcohol exposure does not affect NC induction.

Dysregulation of cell proliferation and apoptosis are implicated in alcohol-triggered neural crest deficiency [12,20,21]. However, results from different species are controversial and inconsistent [22]. We examined this issue in *Xenopus* model system using immunohistochemistry with phosphohistone H3 (pH3) and activated caspase-3 antibodies to detect cell proliferation and apoptosis, respectively. The Xenopus embryos were incubated in alcohol-MBS buffer at stage 9 until harvest time at stage16. Alcohol exposure at 2% did not change the number of pH3 positive cells in the NC region at stage 16 (Figure 2D pink cells inside white dot line box). The cell apoptosis was not increased at stage 16 until the alcohol concentration over 2% as shown by activated-caspase 3 western blot assay (Figure 2E). Our data suggest that alcohol exposure (<2%) does not affect cell proliferation nor apoptosis in Xenopus neural crest induction.

### Alcohol impairs NC cell migration

**Figure 3:**
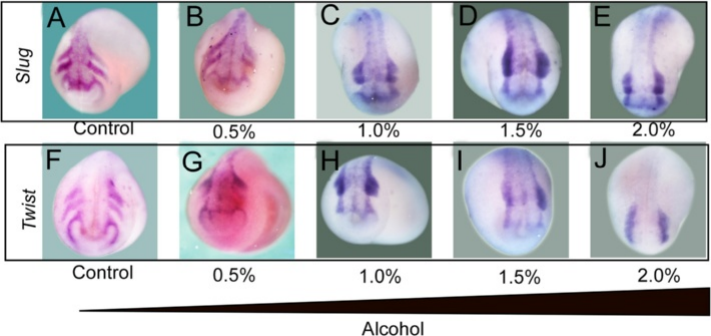
**Alcohol exposure disrupts neural crest migration. (A)** Both slug and twist1 in situ hybridization, performing along with gradient alcohol treatment, displays neural crest migration is accordingly blocked by the increasing concentration of alcohol. **(B,G)** indicates 0.5% alcohol treatment still allows fairly migration of neural crest versus control embryos **(A,F)**. 1.0% alcohol treatment deteriorate the egressing process of neural crest, however, there is perceivable migration occurring **(C,H)**. Finally, both 1.5% and 2.0% appears completely freezing the migration **(D,E,I,J).**

Next we examined whether alcohol exposure affects NC cell migration. The embryos from stage 9 were treated with alcohol and washed at stage 16, and then cultured in MBS until tail bud stage. An *in situ* hybridization assay was performed to determine the expression of migrating marker genes of NC cells-*Twist* and *Slug. Twist* and *Slug* positive cells migrated ventrally in several streams into the hyoid and branchial arches of the *Xenopus* embryo. In control embryos, *Twist* (Figure 3A) and *Slug* (Figure 3F) were expressed in distally migrating NC cells. These markers were confined to much more dorsal regions of the embryo in alcohol exposure groups. This result suggest a decrease in the migration distance that the separated NC streams had moved. In addition, NC cell migration was impaired by alcohol exposure in a dosage dependent manner (Figure 3B-E, G-J). With 2% alcohol treatment, NC cells failed to migrate ventrally into separate streams and were clustered and retained in a dorsal position (Figure 3E, J).

### 5MTHF rescues alcohol-induced neural crest migratory abnormality in Xenopus model

**Figure 4:**
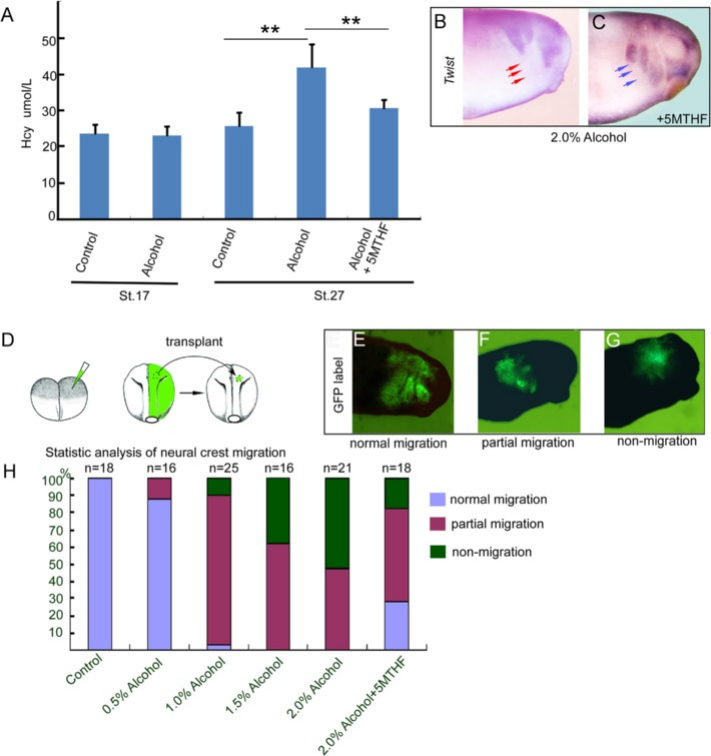
**5-mehtyltetrahydrofolate improves alcohol-induced developmental abnormalities.** **(A)** Homocysteine was significantly increased at stage 27 (from 25.5 ± 3.7 micromole/L /100embryos in control group to 41.8 ± 6.3 micromole/L /100embryos in alcohol group, P < 0.05), whereas the alteration is undetectable statistically at stage 16(23.5 ± 2.5 micromole/L /100embryos in control and 23.0 ± 2.3 micromole/L /100embryos in alcohol group, P > 0.05). Supplementation of 5MTHF significantly (P < 0.05) buffers the accumulation of endogenous homocysteine to 30.5 ± 2.3 micromole/L /100embryos at stage 27. **(B,C)** Injection of 5MTHF restored neural crest migration, and alleviated the effect of alcohol on neural crest migration. **(D)** Schematic diagram illustrating the xenografting experiments. **(E-G)** Alcohol exposure partially or completely blocked GFP-labeled neural crest xenografting migrating in living embryos. **(H)** Quantification of the GFP-labeled Neural crest migation upon gradient alcohol exposure and 5MTHF treatment.

Previous experiments have shown that alcohol consumption decreased the ability of folic acid uptake [23] and accumulated homocysteine in rodent model and human [24,25]. Furthermore, homocysteine has been found to block NC migration [19]. Interestingly, folic acid was found to reverse alcohol induced-teratogenesis in a mouse model [17,18]. In addition, our recent data indicated that folic acid was involved in NC migration [11]. The inhibition of NC migration by alcohol could be attributed to SAM-homocysteine dysregulation. To examine this issue, embryonic homocysteine levels were first evaluated under a 2% alcohol exposure from Xenopus development stage 9. At the NC pre-migratory time (stage 16), alcohol exposure did not significantly change the amount of endogenous homocysteine (23.5 ± 2.5 μM in control and 23.0 ± 2.3 μM in alcohol, P > 0.05). However, alcohol treatment clearly increased homocysteine levels at stage 27 when the NC cells underwent robust emigration (25.5 ± 3.7 μM in control and 41.8 ± 6.3 μM in alcohol, P < 0.05) (Figure 4A). Microinjection of 5MTHF, the most bioactive form of folic acid, attenuated alcohol-induced elevations of homocysteine levels (30.5 ± 2.3 μM) (Figure 4A). To determine whether 5MTHF alleviated the effect of alcohol on NC migration, the embryos were injected with 5MTHF at two cell stages prior to being immersed in the alcohol-containing MBS buffer. The neural crest migration was analyzed with an *in situ* hybridization assay of *Twist* expression at the tadpole stage. *Twist* positive cells, absent in the most ventral region of branchial arch upon alcohol exposure (Figure 4B), were clearly present in most of the 5-MTHF injected embryos (Figure 4C). To further exclude that the absence of *Twist* expression was not due to any disabled differentiation processes (i.e. the inability of twist expression to further induce cellular differentiation) during the migration of the NC cells, embryos were first labeled by injection of a GFP mRNA into one blastomere at the two cell stage. The GFP-positive neural crest cells from the injected blastomere were dissected from the donor embryos and transplanted into the similar place of normal embryos at the neurula stage (Figure 4D). In MBS control groups (n = 18), these cells were found to migrate ventrally in three fluorescent streams (Figure 4E). However, alcohol incubation at the beginning of gastrulation to NC transplant time (stage 16) of donor embryos partially or completely impaired the ventral movement of the fluorescent streams (Figure 4F, G). Upon 0.5% alcohol exposure, 12.5% fluorescent graft exhibited partial migration (n = 16). At 1% alcohol concentration, most of the grafts (22/25) migrated partially and 2 grafts exhibit non-migration. The percentage of migration impairment was increased up to 62.5% partial migration, 37.5% non-migration (n = 16) at 1.5% alcohol exposure. 2% alcohol exposure resulted in 47.6% partial migration and 52.4% non-migration (n = 21) (Figure 4H) and this migratory impairment was alleviated to 55.5% partial migration and 16.7% non-migration with 27.8% (n = 18) showing normal migration when the donor embryos were injected with 5-MTHF at the 2 cell stage (Figure 4H). These results suggested that alcohol affected folic acid-homocysteine metabolism and thereby disrupted the neural crest migration in *Xenopus* model.

FASD has been largely attributed to dysregulation of NC development via either disruption of migration or cell apoptosis. In this study we found that alcohol treatment at 2% triggers apoptosis in Xenopus embryos, leading to neural crest deformations. Interestingly, we found that both 1.5% and 1.0% specifically inhibited NC migration without detectable apoptosis. These results indicate that alcohol preferentially affects the initiation of NC migration. Moreover, we found that the process of NC induction was not affected at 1.5% and 1.0% of alcohol treatment, suggesting that alcohol specifically interferes with NC migration. Our study suggest that human FADS could be more related to defective NC migration than apoptosis since human blood alcohol concentration is usually less than 0.5%.

FASD is a developmental disorder and the interaction between environment and gene plays a major role in the pathogenesis of FASD [26,27]. Our study showed that 5-MTHF could potentially prevent the alcohol-induced developmental abnormalities. 5-MTHF plays a critical role in nucleotide synthesis and methylation processes. 5-MTHF is important to recycle homocysteine and synthesize S-adenosylmethionine (SAM). SAM is the main methyl donor providing methyl residue for the most of biological methylation reactions [28]. Our previous work has indicated that modulating the metabolism of 5-MTHF affected the histone 3 lysine (H3K) methylation during neural crest development [11]. Furthermore, it has been reported that the H3K methylation preferentially takes place at *Snail* and *Twist1* enhancer, consequently controlling neural crest migration by altering transcriptional accessibility [29]. Our study showed that alcohol resulted in accumulation of homocysteine at relatively late stage, indicating decreased level of 5-MTHF [30-32] during early neural crest development. Addition of 5-MTHF enables neural crest the capability of blocking the detrimental effects of alcohol on development. Clinical studies also showed significantly enhancement of serum homocysteine level upon alcohol consumption [24,25]. Homocysteine has been known as a risk factor for neurocristopathies [33-36]. Alcohol appears to perturb neural crest migration by obstructing 5-MTHF absorption and increasing homocysteine. These detrimental effect could result in the abnormalities in FASD. Thus, modulation of one carbon cycle might be beneficial to prevention of FASD.

During embryonic development, alcohol targets many types of cell and/or organ [37-41]. Importantly, our observations are consistent with previous studies that alcohol causes NC migratory defectives via promoting apoptosis. Meanwhile, our results further indicate that the dysregulation of one carbon cycle during NC migration could be a novel mechanism underlying FASD. This discovery shed a light on the therapeutic possibility of folic acid. Our experiments were specifically designed to investigate toxicity of alcohol on NC development. Mesoderm plays a center role from the initiation to the migration of NC development, and tightly regulates the formation of whole body plan. Thus, further study is warranted to examine the role of mesoderm in NC development and its effect on FASD pathogenesis.

## Conclusions

Our study demonstrate that prenatal alcohol exposure causes neural crest cell migration abnormality and 5-mehtyltetrahydrofolate could be beneficial for treating fetal alcohol spectrum disorders.

## Methods

### Xenopus experiments

The experimental procedures for *in vitro* fertilization, manipulation and microinjection of the Xenopus embryos, as well as for the *in situ* hybridization of whole mount embryo were previously described [42]. The cartilage staining was carried out using Alcian blue 8GX according to protocol of Dr. Richard Harland’s lab (http://tropicalis.berkeleyedu/home/gene_expression/cartilage-stain/alcian.html) as well as described [43] and the *in situ* probes for *Zic1*, *Pax3 Slug* and *Twist* gene expression were described previously [44]. Microinjection was performed using the PLI-1 Pico-injector (Harvard Apparatus) equipped with the MK-1 micromanipulator (Singer Instruments).

### Reverse transcription - polymerase chain reaction (RT-PCR)

To test the effect of alcohol on neural crest induction, RT-PCR was carried out using whole embryos at the onset of gastrulation (stage9) until stage 16. H4 was used as a loading control. The primers used for PCR were: Pax3: 5′-CAGCCGAATTTTGAGGAGCAAAT-3′ and 5′-GGGCAGGTCTGGTTCGGAG TC-3′; Snail2: 5′ -TCC- CGCACTGAAAATGCCACGATC -3′ and 5′- CCGTCCTAA- AGATGAAGGGTATCCTG -3′. The primers for Msx1 were used as described [11]. Please simply describe the procedure.

### Embryo immunohistochemistry and western blot

Embryos were collected at stage 16 and fixed with paraformaldehyde. The frozen samples were sectioned in 10 μm and the sections were stained with 1:200 Anti-phospho-histone H3 (Millipore 16–657) to detect cell proliferation and 300 nM DAPI (Life Technologies D1306) for nuclear conterstain. Whole embryos at stage 16 were lysed with 1%triton-X100 in PBS containing a protease inhibitor cocktail (Roche) and the samples were subject to Western blot analysis with activated caspase3 antibody(Abcam 13847) to check cell apoptosis.

### Homocysteine measurement

One hundred *Xenopus* embryos were collected at either stage 16 and 27 and homogenized in 500 μl of lysis buffer [1%triton-X100 in PBS containing a protease inhibitor cocktail (Roche)]. The samples were centrifuge at 12000 g for 10 min at 4°C. After centrifugation, samples were separated into 3 phases; the lower sediment, intermediate water phase (which contained homocysteine) and upper lipid phase. The intermediate water phase was carefully collected without any lipid contamination. The homocysteine concentration was determined by an enzymatic method (produced by Zhongyuan Bio. Ltd China) on an automatic analyzer (Olympus AU 2700). The linear range of Hcy measurement is from 3 μM to 50 μM.

### Statistical analysis

All experiments were repeated at least 3 times, and the P value were calculated with T-TEST.
